# What is the impact of human leukocyte antigen mismatching on graft survival and mortality in renal transplantation? A meta-analysis of 23 cohort studies involving 486,608 recipients

**DOI:** 10.1186/s12882-018-0908-3

**Published:** 2018-05-18

**Authors:** Xinmiao Shi, Jicheng Lv, Wenke Han, Xuhui Zhong, Xinfang Xie, Baige Su, Jie Ding

**Affiliations:** 1 Department of Pediatrics, Peking University First Hospital, Beijing, China; 2 Renal Division, Peking University First Hospital, Beijing, China; 3 Peking University Institute of Nephrology, Beijing, China; 4 Key Laboratory of Renal Disease, Ministry of Health of China, Beijing, China; 5 Key Laboratory of Chronic Kidney Disease Prevention and Treatment, Peking Unversity, Ministry of Education, Beijing, China; 6 Institute of Urology, Peking University, Beijing, China; 7 Department of Urology, Peking University First Hospital, Beijing, China

**Keywords:** Human leukocyte antigen, Kidney transplantation, Graft survival, Mortality, Meta-analysis

## Abstract

**Background:**

The magnitude effects of human leukocyte antigen (HLA) mismatching on post-transplant outcomes of kidney transplantation remain controversial. We aim to quantitatively assess the associations of HLA mismatching with graft survival and mortality in adult kidney transplantation.

**Methods:**

We searched PubMed, EMBASE and the Cochrane Library from their inception to December, 2016. Priori clinical outcomes were overall graft failure, death-censored graft failure and all-cause mortality.

**Results:**

A total of 23 cohort studies covering 486,608 recipients were selected. HLA per mismatch was significant associated with increased risks of overall graft failure (hazard ratio (HR), 1.06; 95% confidence interval (CI), 1.05–1.07), death-censored graft failure (HR: 1.09; 95% CI 1.06–1.12) and all-cause mortality (HR: 1.04; 95% CI: 1.02–1.07). Besides, HLA-DR mismatches were significant associated with worse overall graft survival (HR: 1.12, 95% CI: 1.05–1.21). For HLA-A locus, the association was insignificant (HR: 1.06; 95% CI: 0.98–1.14). We observed no significant association between HLA-B locus and overall graft failure (HR: 1.01; 95% CI: 0.90–1.15). In subgroup analyses, we found recipient sample size and ethnicity maybe the potential sources of heterogeneity.

**Conclusions:**

HLA mismatching was still a critical prognostic factor that affects graft and recipient survival. HLA-DR mismatching has a substantial impact on recipient’s graft survival. HLA-A mismatching has minor but insignificant impact on graft survival outcomes.

**Electronic supplementary material:**

The online version of this article (10.1186/s12882-018-0908-3) contains supplementary material, which is available to authorized users.

## Background

Compared with dialysis, renal transplantation is a more preferred option for end-stage renal disease (ESRD) [1]. In recent report of global database on donation and transplantation (http://www.transplant-observatory.org), about 80,000 renal transplants were performed annually [2]. However, in 2016 United States Renal Data System (USRDS) Annual Data Report, the long-term survival benefit remained unsatisfactory, with ten-year graft survival probabilities of 46.9% for deceased donor transplant [3].

Human leukocyte antigen (HLA) was important biological barrier to a successful transplantation and has substantial impact on the prolongation of graft survival [4]. However, the emergency of modern immunosuppressive agents minimized the effect of HLA compatibility. The US kidney allocation system was extensively modified to eliminated HLA-A similarity in 1995 [5] and HLA-B similarity in 2003 [6]. In the revised United Kingdom kidney allocation scheme, HLA-A matching is no longer considered [7]. But the latest European Renal Best Practice Transplantation Guidelines still recommended that matching of HLA-A, -B, and -DR whenever possible, while gave more weight to HLA-DR locus [8]. So far, the current kidney allocation guideline recommendations were inconsistent in term of HLA compatibility. Besides, for the primary aim to make the kidney last as long as possible, all the current kidney allocation systems were not perfect. Here, we sought to conduct a meta-analysis to assess the magnitude effect of HLA mismatching in adult kidney transplantation, with a particular focus on graft survival and recipient mortality.

## Methods

The study was registered in the PROSPERO international prospective register of systematic reviews (CRD42017071894). Details of protocol are described in Additional file 1: Supplemental Methods. The meta-analysis was performed in accordance with the Meta-analysis of Observational Studies in Epidemiology (MOOSE) protocol [9] (Additional file 2: Table S1) and the Preferred Reporting Items for Systematic Reviews and Meta-Analysis (PRISMA) guideline [10] (Additional file 3: Table S2).

### Literature search strategy

We searched PubMed, EMBASE and the Cochrane Library from their inception to December, 2016, without language restriction. We used the following combinations of Medical Subject Heading (MeSH) terms and corresponding text words: “kidney transplantation”, “renal transplantation”, “human leukocyte antigen”, “HLA” and all possible spellings of “survival”. Further details are described in Additional file 1. Reference lists of articles were manually screened to identify further relevant studies. The literature search was performed independently by two investigators (XMS and XHZ). Differences were resolved by consensus.

### Study selection

We included studies that (1) included a study cohort comprising adult post-kidney transplant recipients; (2) were cohort studies/trials reporting associations between HLA mismatching and post-transplant survival outcomes; and (3) provided effect estimates of hazard ratios (HRs) with 95% confidence interval (CIs). Studies reporting data on children or animals or in vitro research were excluded. Besides, reviews, meta-analyses, case reports, case series and technical descriptions with insufficient data or unrelated topics were also excluded. For studies covered overlapping data, we included the most recent and informative one. XMS and XHZ independently screened the titles and abstracts for eligibility. Discrepancies were resolved by consensus.

### Outcome measures

Our primary clinical endpoint was overall graft failure; secondary clinical endpoints were death-censored graft failure and all-cause mortality. The European Renal Best Practice Transplantation Guidelines and Kidney Disease: Improving Global Outcomes Guidelines was used to evaluate the incidence of measured outcomes [11, 12].

### Data extraction and quality assessment

Data were extracted from predefined protocol, then recorded in a standardized Excel form, including the first author’s name, publication date, study location, study design, cohort size, recipient age, sex distribution, duration, donor source, data source (multi-centered or single-centered), follow-up, unadjusted and adjusted HRs of overall graft failure, death-censored graft failure and all-cause mortality per HLA-mismatch increased, and adjusted covariates in reported multivariable analysis. We contacted libraries abroad or corresponding author of relevant articles by email when detailed data for pooling analysis was unavailable. The methodological quality of included studies was described using the Newcastle-Ottawa Scale. High-quality studies were defined by a score of > 5 points [13]. Disagreements in the scores were resolved by consensus between XMS, XHZ and JD.

### Statistical analysis

Hazard ratios (HRs) with corresponding 95% confidence intervals (CIs) were directly retrieved from each study. We chose HRs as the statistic estimates because they correctly reflect the nature of data and account for censoring. Cochran’s Q test and *I*^*2*^ statistic were applied to assess heterogeneity between studies. The following criteria were used: *I*^*2*^ < 50%, low heterogeneity; 50–75%, moderate heterogeneity and > 75%, high heterogeneity [14, 15]. When significant heterogeneity was found between studies (*P* < 0.10 or *I*^*2*^ > 50%), the effect estimates were calculated using a random-effects model and the DerSimonian-Laird method [16]; otherwise, a fixed-effects model with the Mantel-Haenszel method was used [17]. Subgroup analyses included recipient sample size (≥10,000 vs < 10,000), the nature of data (univariable-unadjusted vs multivariable-adjusted effect estimates), donor source (deceased vs living and deceased), data source (multi-centered vs single-centered) and geographical locations (Europe, North America, Asia and Oceania). A sensitivity analyses was performed by omitting one study at a time and then reanalyzing the data to assess the change in effect estimates. To further explore heterogeneity, a random-effects univariate meta-regression was conducted when at least 10 studies were available. For outcomes of at least 10 studies included, publication bias was assessed by funnel plot and Egger test [18]. Egger test with two-sided *P* < 0.10 was considered to be statistically significant. Analyses were performed using STATA software, version 13.0 (STATA Corporation, College Station, Texas, USA).

## Results

**Fig. 1:**
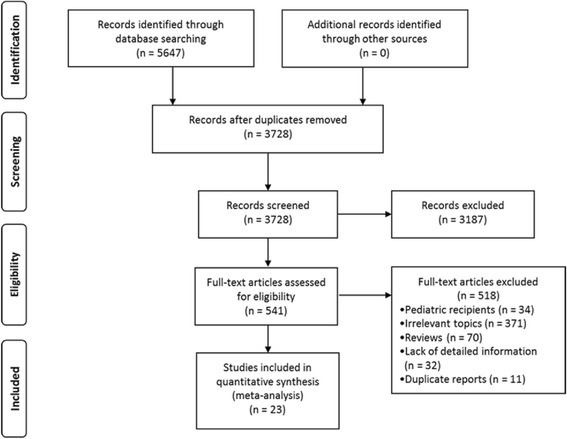
PRISMA flow chart of study selection

Of 5647 articles identified, we reviewed the full text of 541 reports, and 23 studies [19–41] with 486,608 adult post-transplant recipients were included in the meta-analysis (Fig. 1). Detailed characteristics of included studies are presented in Table 1. Among these studies, 18 studies provided multivariable-adjusted effect estimates [19, 21–24, 27–39], 3 studies provided both multivariable-adjusted and univariable-unadjusted data [20, 25, 26], and 2 studies provided univariable-unadjusted data [40, 41]. Besides, 8 studies were multi-centered [19, 23, 27, 32, 33, 35, 37, 38]; another 15 studies were single-centered [20–22, 24–26, 28–31, 34, 36, 39–41]. When considering HLA locus as categories, 11 studies reported survival outcomes of all HLA locus (HLA-A, -B and -DR) [20, 23–28, 32, 35, 36, 40], 8 [19, 20, 22, 29–31, 33, 41] reported HLA-DR locus, 4 with HLA-B locus [19, 30, 31, 33], and 3 with HLA-A locus [30, 31, 33]. The methodological quality score was high, ranging from 6 to 8 points (details of quality assessment are provided in Additional file 4: Table S3).

**Table 1:** Baseline characteristics of the studies included in the meta-analysis

Author, year	No. of recipients	Mean age, years	Male, %	Year data collection	Country of origin	Data Source	Type of risk (Adjustments)	Quality score
De Fijter, 2001 [20]	496	47.4	62.1	1983–1997	Netherlands	Single-center	Recipient and donor age and gender, CIT, PRA, initial immunosuppression, DGF, ARE, type of AR	8
Roodnat, 2003 [21]	1124	44.8	58.5	1981–2000	Netherlands	Single-center	Recipient and donor age, donor gender, CIT, donor Cr, transplantation year, donor type, number of previous transplants	8
Tekin, 2015 [22]	2633	47.7	40.4	2008–2013	Turkey	Single-center	Recipient and donor age and gender, donor follow-up Cr levels, time on dialysis, original disease, CIT, DGF, ARE, recipient serum Cr-levels, warm ischemia times	7
Mandal, 2003 [23]	31,909	NR	59.0	1995–1998	USA	Registry (USRDS)	Recipient and donor age, recipient gender and race, donor type, CIT, diabetic nephropathy	8
Arias, 2007 [24]	214	47.7	64.0	NR	Spain	Single-center	Recipient and donor age and gender, donor type, ARE, CMV, CIT, PRA, glomerulosclerosis, interstitial fibrosis, tubular atrophy, arteriosclerosis, arteriolar hyalinosis	8
Cho, 2016 [25]	229	63.2	63.6	1995–2014	Korea	Single-center	Recipient and donor age, donor type, recipient gender, ABO-incompatible, DGF, CMV, HBV, HCV, time on dialysis prior to transplantation	8
Gomez, 2013 [26]	487	38.0	63.2	1979–1997	Spain	Single-center	Recipient and donor age, donor gender, donor type, DGF, CIT, PRA, AR, time on dialysis, immunosuppression	8
Laging, 2012 [28]	1821	47.8	62.0	1990–2009	Netherlands	Single-center	Recipient and donor age, maximum PRA, current PRA, transplant year, donor gender, donor type, DGF, immunosuppression	8
Laging, 2014 [35]	1998	48.2	62.5	1990–2010	Netherlands	Single-center	Recipient and donor age, donor gender, donor type, PRA, transplant year, immunosuppression	8
Schnuell*e*, 1999 [30]	152	46.4	56.7	1989–1998	Germany	Single-center	Recipient and donor age, recipient gender, time on dialysis, original disease, PRA, dopamine, noradrenaline, head trauma, previous transplant, immunosuppression, Induction (ATG/OKT3)	8
Hariharan, 2002 [32]	105,742	NR	NR	1988–1998	USA	Registry (OPTN/UNOS)	Recipient and donor age and race, gender, DM, hypertensive nephropathy, pre-TX dialysis and transfusions, previous transplant, most recent PRA, DGF, donor type, 1-year AR, induction therapy, immunosuppression regiment	7
Massie, 2016 [19]	106,019	50.0	62.4	2005–2013	USA	Registry (SRTR)	Recipient and donor age, gender and race, PRA, transplant year, private insurance, HCV, eGFR, BMI, cigarette use, SBP, ABO-incompatible, unrelated to recipient, min(donor/recipient weight ratio,0.9)	8
Cho, 2012 [33]	39,332	52.0	51.2	2000–2008	USA	Registry (OPTN/UNOS)	Recipient and donor age, gender, race, CAD, CVD, DM, PVD, pulmonary, malignancy, CMV, DGF, rejection treatment	8
Croke, 2010 [27]	12,662	NR	NR	1985–2007	Australia/ New Zealand	Registry (ANZDATA)	Donor and recipient variables,transplant year,type of initial CNI	8
Connolly, 1996 [31]	516	NR	67.4	1989–1993	UK	Single-center	Recipient and donor age and gender, donor type, DGF, CIT, PRA, ARE, warm ischemia time,	8
Asderakis, 2001 [29]	788	42.1	67.8	1990–1995	UK	Single-center	Recipient and donor gender, donor age, DGF, CIT, ARE, immunosuppression	8
Opelz, 2007 [35]	135,970	NR	61.6	1985–2004	Germany	Registry (CTS)	Recipient and donor age, gender, and race, PRA, CIT, transplant year, time on dialysis, original disease, previous transplant, pre-transplant dialysis, recipient geographical origin, immunosuppression	8
Amatya, 2010 [36]	229	40.5	59.4	1997–2007	USA	Single-center	Recipient age, gender, and race, BMI, PRA, previous transplant, CIT, WIT	8
Zukowski, 2014 [40]	232	37.7	63.8	1997–1998	Poland	Multi-center	Univariate	6
Fellstrom, 2005 [41]	2102	51	65.2	1996–1997	Europe	Multi-center	Univariate	6
Summers 2010 [37]	9134	47	61.4	2000–2007	UK	Registry (UK transpalnt registry)	Recipient age, donor age, CIT	6
Van 1996 [39]	1289	32.8	60.9	1966–1994	Netherlands	Single-center	Recipient and donor age and gender, type of immnnosuppression, presensitisation, DM, and living or post-mortem donor	8
Lynch 2013 [38]	31,534	N/A	N/A	2000–2010	USA	Registry (SRTR)	Not reported	8

*ANZDATA* Australia and New Zealand Dialysis and Transplant Registry, *AR* acute rejection, *ARE* acute rejection episode, *ATG* antithymocyte globulin, *BMI* body mass index, *CAD* coronary artery disease, *CIT* cold ischemic time, *CMV* cytomegalovirus, *CNI* calcineurin inhibitor, *Cr* creatinine, *CTS* Collaborative Transplant Study, *CVD* cardiovascular disease, *DGF* delayed graft function, *DM* diabetes mellitus, *GFR* glomerular filtration rate, *HBV* hepatitis B virus, *HCV* hepatitis C virus, *NR* not reported, *OPTN* the Organ Procurement and Transplantation Network, *PRA* panel reactive antibodies, *pre-TX* pre-transplant, *PVD* peripheral vascular disease, *SBP* systolic blood pressure, *SRTR* Scientific Registry for Transplant Recipient, *UNOS* United Network for Organ Sharing, *USRDS* United States Renal Data System, *WIT* warm ischemic time

### Primary outcomes

#### HLA per mismatch and overall graft failure

**Fig. 2:**
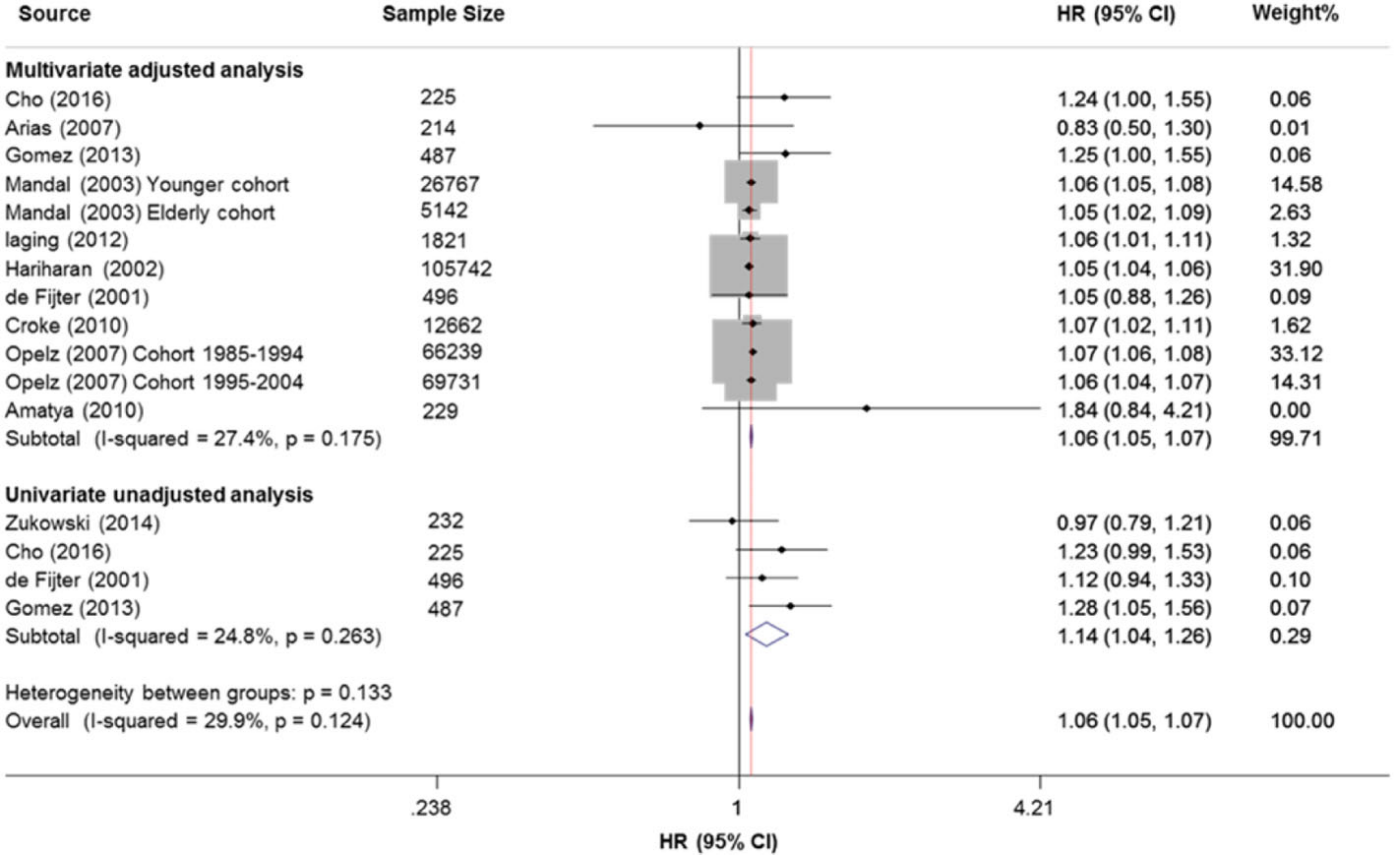
Forest plots of the association between HLA per mismatch and overall graft failure, using both of univariable-unadjusted and multivariable-adjusted effect estimates

Eleven studies (289,987 adult recipients) reported data on HLA mismatching and overall graft failure. The pooled analysis revealed that each incremental increase of HLA-mismatches was significant associated with a higher risk of overall graft failure, both in univariable-unadjusted summary estimates (HR: 1.14; 95% CI: 1.04–1.26; *P* = 0.008; Fig. 2) and multivariable-adjusted summary estimates (HR: 1.06; 95% CI: 1.05–1.07; *P* < 0.001; Fig. 2). The heterogeneity was low (*I*^*2*^ = 24.8 and 27.4%, respectively). Detailed predefined subgroup analyses were listed in Table 2. The effect estimates did not changed significantly after stratification for sample size (≥10,000 vs < 10,000), data source (multi-centered vs single-centered), donor source (cadaveric vs living and cadaveric), geographic locations (European, North America, Asia and Oceania) and year period (prior to 1995 vs not prior to 1995). In sensitivity analysis, the summary estimates were not modified after excluding one study at a time. Subsequent univariate meta-regression indicated that these factors did not significantly change the overall effect (Additional file 5: Fig. S1). Publication bias was not significant (Additional file 6: Fig. S2).

**Table 2:** Subgroup analyses of overall graft failure associated with HLA per mismatch and HLA-DR mismatches

	HLA per mismatch				HLA-DR mismatches			
Subgroup	No. of recipients (cohorts)	HR (95% CI)	*I* ^*2*^	*P* ^a^	No. of recipients (cohorts)	HR (95% CI)	*I* ^2^	*P* ^a^
Sample size				0.835				0.011
≥10,000	281,141 (5)	1.06 (1.05–1.07)	49.1		145,351 (2)	1.07 (1.04–1.10)	0	
< 10,000	8614 (7)	1.06 (1.03–1.09)	17.3		4652 (5)	1.27 (1.12–1.43)	48.4	
Nature of data				0.133				0.076
Univariable-unadjusted	1440 (4)	1.14 (1.04–1.26)	24.8		2598 (2)	1.39 (1.05–1.83)	70.0	
Multivariable-adjusted	286,755 (12)	1.06 (1.05–1.07)	27.4		150,003 (7)	1.08 (1.05–1.11)	58.3	
Data source				0.683				0.011
Registry/Multi-center	286,283 (6)	1.06 (1.05–1.07)	38.9		145,351 (2)	1.07 (1.04–1.10)	0	
Single-center	3472 (6)	1.07 (1.02–1.12)	26.6		4652 (5)	1.27 (1.12–1.43)	48.4	
Donor source				0.027				0.616
Cadaveric	138,516 (5)	1.07 (1.06–1.07)	0.00		41,284 (5)	1.08 (1.04–1.11)	66.4	
Living and Cadaveric	151,239 (7)	1.05 (1.05–1.06)	16.4		108,719 (2)	1.09 (1.04–1.15)	55.4	
Geographical locations				0.053				0.033
Europe	138,988 (6)	1.07 (1.06–1.08)	0.00		1952 (4)	1.32 (1.09–1.60)	59.7	
North America	137,880 (4)	1.05 (1.04–1.06)	2.1		145,351 (2)	1.07 (1.04–1.10)	0	
Asia	225 (1)	1.24 (1.00–1.55)	–		2700 (1)	1.23 (1.05–1.45)	–	
Oceania	12,662 (1)	1.07 (1.03–1.12)	–		0	–	–	
Year period				0.175				0.026
Prior to 1995	103,915 (6)	1.06 (1.05–1.07)	0.00		148,051 (3)	1.08 (1.04–1.12)	26.8	
Not prior to 1995	185,626(5)	1.06 (1.05–1.08)	60.1		4054 (4)	1.36 (0.98–1.88)	59.7	
NR	214 (1)	0.83 (0.51–1.34)	–	–	–	–	–	–

The effect estimates were stratified for sample size (≥10,000 vs < 10,000), data source (multi-centered vs single-centered), donor source (cadaveric vs living and cadaveric), geographic locations (European, North America, Asia and Oceania) and year period (prior to 1995 vs not prior to 1995)

^a^*P* value for heterogeneity

#### HLA-DR mismatches and overall graft failure

**Fig. 3:**
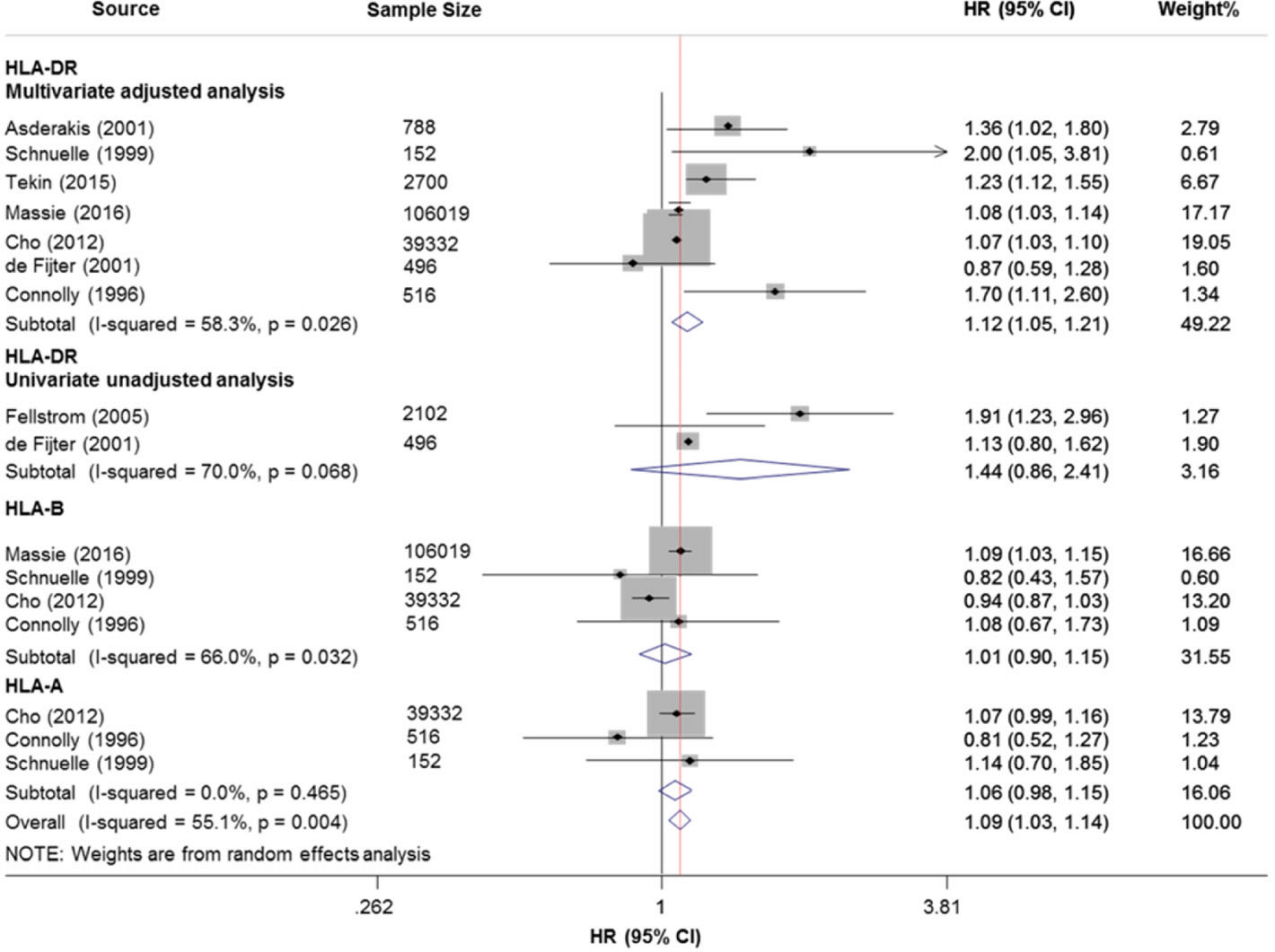
Forest plots of the association between HLA-A, -B, -DR mismatches and overall graft failure

Eight studies with 152,105 adult recipients were analyzed to investigate the association between HLA-DR mismatching and overall graft failure. The pooled results revealed an unadjusted HR of 1.44 (95% CI: 0.86–2.41; *P* = 0.160) with moderate heterogeneity (*I*^*2*^ = 70.0%). After adjustment, each incremental increase of HLA-DR mismatches was significant associated with 12% higher risk of overall graft failure (HR: 1.12; 95% CI: 1.05–1.21; *P* = 0.002; Fig. 3), with moderate heterogeneity (*I*^*2*^ = 58.3%). Sensitivity analysis with a fixed-effects model obtained similar results (HR: 1.08; 95% CI: 1.05–1.11; *P* < 0.001). Subsequent subgroup analysis demonstrated that the effect was not modified after stratification for sample size, data source, donor source and geographical locations (Table 2). The effect estimates remained stable after excluding one study at a time. Considering only 8 studies included in meta-analysis, we did not perform a meta-regression.

In addition, three studies with 41,957 recipients evaluated 1 or 2 DR-mismatches versus 0 DR-mismatches. Compared with 0 mismatches in HLA-DR antigen, 1 mismatches and 2 mismatches were all associated with higher risk of overall graft failure, with pooled HRs of 1.12 (95% CI: 1.04–1.21; *P* = 0.002) and 1.15 (95% CI: 1.05–1.25; *P* = 0.002), respectively (Additional file 7: Fig. S3). In both pooled analysis, there was no heterogeneity (*I*^*2*^ = 0%).

#### HLA-B mismatches and overall graft failure

Associations of HLA-B epitope and overall graft failure were reported in 4 studies with 146,019 recipients. The pooled analysis demonstrated that each incremental increase of HLA-B mismatches was not associated with higher risk of overall graft failure (HR: 1.01; 95% CI: 0.90–1.15; *P* = 0.834; Fig. 3), with moderate heterogeneity (*I*^*2*^ = 66.0%). Sensitivity analysis with a fixed-effects model obtained similar effect estimates (HR: 1.01; 95% CI: 0.89–1.14; *P* = 0.079). In addition, the effect estimates did not changed significantly after stratification for sample size (≥10,000 vs < 10,000) of cohorts.

#### HLA-A mismatches and overall graft failure

Only 3 studies (40,000 recipients) reported data on the association of HLA-A epitope and overall graft failure. The results revealed an insignificant association (HR: 1.06; 95% CI: 0.98–1.14; *P* = 0.121; Fig. 3), with no heterogeneity (*I*^*2*^ = 0%). Sensitivity analysis with a random-effects model showed similar results (HR: 1.06; 95% CI: 0.98–1.15; *P* = 0.121). The results should be cautiously interpreted because of only three studies included.

### Secondary outcomes

#### Death-censored graft failure

**Fig. 4:**
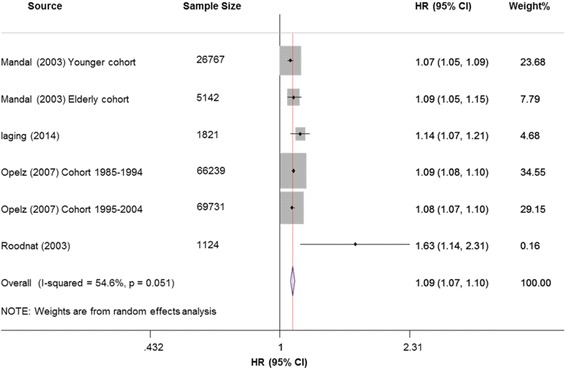
Forest plots of the association between HLA per mismatch and death-censored graft failure

We included 101,093 recipients from 4 cohorts. Each incremental increase of HLA mismatches was associated with a higher risk of death-censored graft failure, with summary HR of 1.09 (95% CI: 1.06–1.12; *P* < 0.001; Fig. 4), and moderate heterogeneity (*I*^*2*^ = 70.9%). Sensitivity analysis with a fixed-effects model showed similar results (HR: 1.09; 95% CI: 1.08–1.10; *P* < 0.001). The summary estimates were not modified after including only large sample size of cohorts (> 10,000 recipients) (Additional file 8: Fig. S4).

#### All-cause mortality

**Fig. 5:**
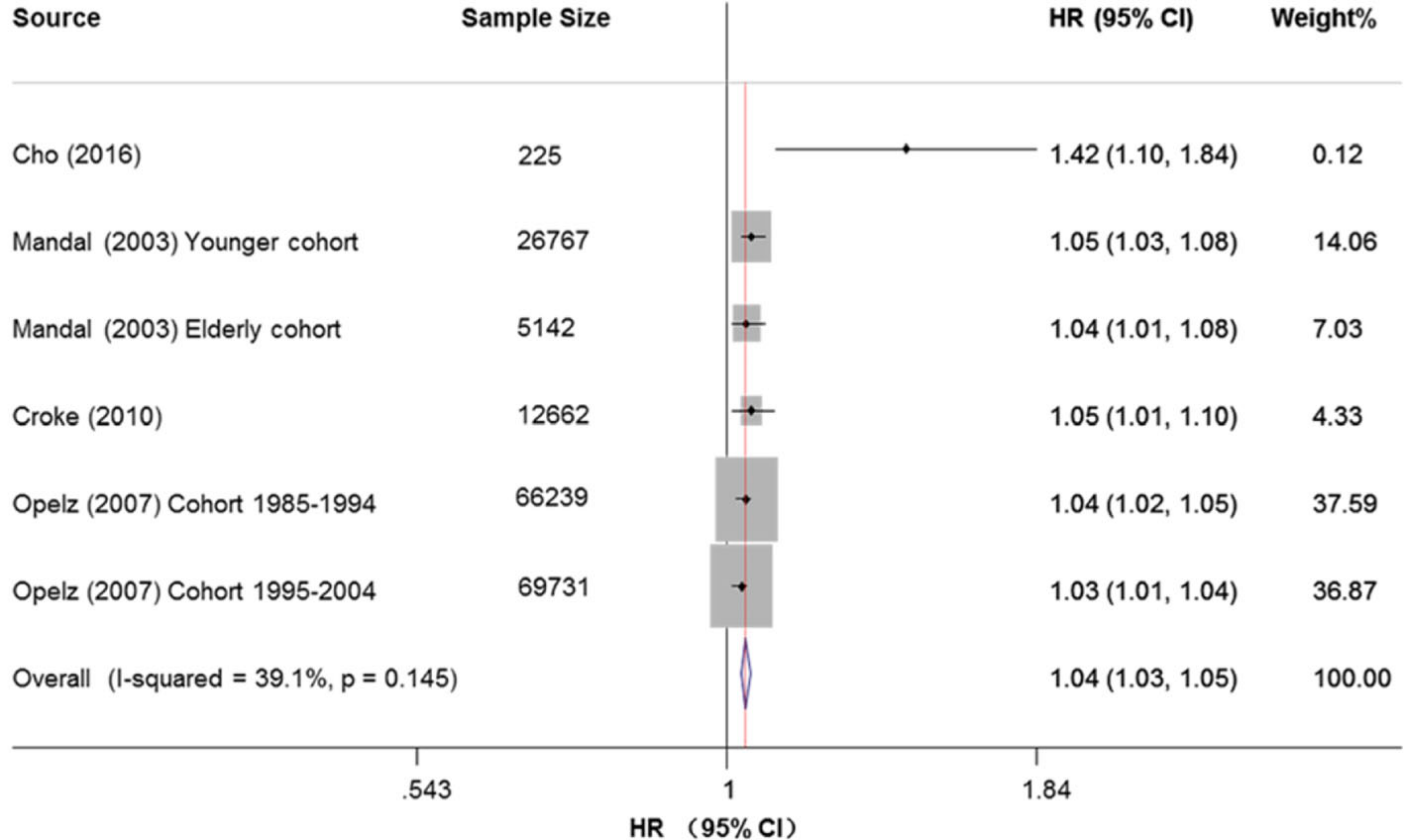
Forest plots of the association between HLA per mismatch and all-cause mortality

We included 180,766 recipients from 4 cohorts. Each incremental increase of HLA mismatches was associated with a higher risk of all-cause mortality rates (HR: 1.04; 95% CI: 1.02–1.07; *P* = 0.001; Fig. 5). The heterogeneity was moderate (*I*^*2*^ = 65.3%). Summary estimates did not changed significantly after analyzing with a fixed-effects model (HR: 1.04; 95% CI: 1.02–1.07; *P* = 0.001). After stratification for sample size of cohorts (≥10,000 vs < 10,000), the effect estimates were not modified (HR: 1.04; 95% CI: 1.02–1.05; *P* < 0.001; *I*^*2*^ = 27.8%, Additional file 8: Fig. S4). However, the results should be cautiously interpreted due to small number of included studies (*n* = 4).

## Discussion

This is the first meta-analysis to evaluate the magnitude effect of HLA mismatching on post-transplant survival outcomes of adult kidney transplantation. The analysis included 23 studies with a large sample of subjects (totally 486,608 recipients). The results indicated that each incremental increase of HLA mismatches was significantly associated with higher risks of overall graft failure, death-censored graft failure and all-cause mortality. The pooled results also indicated that HLA-DR mismatches were significantly associated with a 12% higher risk of overall graft failure. We also observed that HLA-A per mismatch was associated with a 6% higher risk of overall graft failure, but the association was insignificant. There was no significant association between HLA-B mismatching and graft survival. All included studies were in high methodological quality and the heterogeneity between studies was acceptable in each pooling analysis. In addition, we found that sample size or recipient ethnicity may be potential sources of heterogeneity.

Human HLA genes are located on chromosome 6 and code for 3 major class I alleles (HLA-A, -B, -C) and 3 major class II alleles (HLA-DR, -DQ, -DP). Polymorphisms in HLA, especially HLA-A, -B, and -DR loci, are important biological barriers to a successful transplantation [42, 43]. As closely HLA-matched graft is less likely to be recognized and rejected, HLA mismatching has a substantial impact on prolongation of graft survival. With the emergence of potent immunosuppressive agents that steadily improved the graft survival rates, the impact of HLA compatibility seems to be minimized [42, 44]. But the recent Australia and New Zealand Dialysis and Transplant Registry (ANZDTR) survey with 12,662 recipients still demonstrated that each incremental increase of HLA mismatches was significantly associated with higher risk of graft failure and rejection [27]. Another recent survey from Massie et al. [19] with 106,019 recipients from the Scientific Registry for Transplant Recipients (SRTR) database revealed that HLA-B and -DR mismatches were all significant associated with worse graft survival outcomes. Using multivariable-adjusted data (adjusting for other determinant confounders such as donor and recipient age, gender, combined disease, serum creatinine levels, ischemic times, etc.), the present analysis indicated that HLA per mismatch was associated with an increased risk of overall graft failure (9%), death-censored graft failure (6%) and all-cause mortality (4%). The pooled results were in favor of recommendations of the latest European Renal Best Practice Transplantation Guidelines, which recommended that matching of HLA-A, -B, and -DR whenever possible [8].

The meta-analysis suggested that HLA-DR per mismatch was significant associated with a 12% higher risk of overall graft failure. Besides, a subsequent analysis suggested that compared with 0 DR-mismatches, 1 and 2 mismatches were significant associated with 12 and 15% higher risk of overall graft failure, respectively. The pooled results were in favor of the kidney allocation guideline recommendations in almost all countries, such as the current US kidney allocation system, the revised United Kingdom kidney allocation scheme, and the latest European Renal Best Practice Transplantation Guidelines, which all highlighted the importance of HLA-DR testing [5–8].

Notably, the present analysis revealed a tendency that HLA-A mismatching had an impact on overall graft survival as there were only 3 studies included with a pooled HR of 1.06 (95% CI: 0.98–1.14). However, we did not observe a significant association between HLA-B mismatching and overall graft survival (HR: 1.01; 95% CI: 0.90–1.15). Our pooled results were inconsistent with the recommendations of the revised United Kingdom kidney allocation scheme, which eliminated the impact of HLA-A similarity instead of HLA-B similarity [7]. Moreover, miscellaneous factors can result in inferior outcomes [45]. For instance, inferior graft outcomes could be related to high risk for rejection particularly antibody-mediated rejection [45–47]. Inferior patient survival could partly be associated with consequences of enhanced immunosuppression [45]. Consequently, the pooled results should be cautiously interpreted and further studies should be conducted to investigate the impact of HLA-A mismatching on graft and recipient survival outcomes.

Subgroup analysis and meta-regression was conducted to explore heterogeneity between studies. In subgroup analysis of the association between HLA per mismatch and overall graft failure, we found that after stratification for donor source (cadaveric vs living and cadaveric), the heterogeneity decreased to insignificant (*I* ^*2*^ = 0 and 16.4, respectively). But subsequent meta-regression analysis revealed that donor source did not change the overall effect significantly. In subgroup analysis of the association between HLA-DR mismatching and overall graft failure, we found that ethnicity and recipient sample size were potential source of heterogeneity. Large sample size of cohorts usually demonstrated more stable results. Besides, ethnic diversity was a potential source of heterogeneity probably because of varying HLA polymorphisms in the genetic makeup of the geographically distinct cohorts.

### Strengths and limitations

Strengths of our meta-analysis are large sample of subjects (totally 486,608 recipients) and strict study design. Besides, we used multivariable-adjusted data for pooling analysis, which adjusted for some primary determinant confounders. However, the present meta-analysis had some limitations. Firstly, the absence of randomized controlled trials was the biggest limitation of this meta-analysis. Secondly, several studies have suggested that other HLA loci, such as HLA-C and -DQ locus, may contribute to poorer graft outcomes [48–50], but this meta-analysis only included the HLA-A, -B and -DR loci. Thirdly, heterogeneity is inevitable in some outcomes. We conducted several subgroup and meta-regression analyses to explore the potential source of heterogeneity, and used random-effects models to incorporate heterogeneity between studies. Fourthly, few studies included could provide data about induction agent, maintenance agent or PRA, so that it cannot be achieved to do the stratified analysis.

## Conclusions

HLA mismatching was still a critical prognostic factor that affects graft and recipient survival. HLA-DR mismatching has a substantial impact on recipient’s graft survival. HLA-A mismatching has minor but not significant impact on graft survival outcomes. Further studies should be conducted to confirm the impact of HLA-A similarity.

## Additional files

Additional file 1: Supplemental Methods. (DOCX 45 kb)

Additional file 2: **Table S1.** The MOOSE checklist. (DOCX 103 kb)

Additional file 3: **Table S2.** The PRISMA checklist. (DOCX 90 kb)

Additional file 4: **Table S3.** Newcastle-Ottawa Scale (NOS) score for evaluation of study quality. (DOCX 73 kb)

Additional file 5: **Figure S1.** Meta-regression of HLA mismatches on graft failure for primary determinant confounders (A: Sample size; B: Data source; C: Donor source; D: Geographical locations). (DOCX 608 kb)

Additional file 6: **Figure S2.** Funnel plot and Egger test for publication bias among studies that evaluated association HLA per mismatch and overall graft failure. (A: Funnel plot; B: Egger test). (DOCX 81 kb)

Additional file 7: **Figure S3.** Forest plot that evaluated the impact of 1 or 2 HLA-DR mismatches versus 0 mismatches on overall graft failure. (DOCX 587 kb)

Additional file 8: **Figure S4.** Forest plot after stratification for sample size (≥10,000 vs < 10,000) of cohorts, to evaluate association between (A) HLA per mismatch and death-censored graft failure; (B) HLA per mismatch and all-cause mortality. (DOCX 1230 kb)

## Abbreviations

ANZDATA: Australia and New Zealand Dialysis and Transplant Registry
AR: Acute rejection
ARE: Acute rejection episode
ATG: Antithymocyte globulin
BMI: Body mass index
CAD: Coronary artery disease
CI: Confidence interval
CIT: Cold ischemic time
CMV: Cytomegalovirus
CNI: Calcineurin inhibitor
Cr: Creatinine
CTS: Collaborative Transplant Study
CVD: Cardiovascular disease
DGF: Delayed graft function
DM: Diabetes mellitus
ESRD: End-stage renal disease
GFR: Glomerular filtration rate
HBV: Hepatitis B virus
HCV: Hepatitis C virus
HLA: Human leukocyte antigen
HR: Hazard ratio
NR: Not reported
OPTN: The Organ Procurement and Transplantation Network
PRA: Panel reactive antibodies
pre-TX: Pre-transplant
PVD: Peripheral vascular disease
SBP: Systolic blood pressure
SRTR: The Scientific Registry for Transplant Recipient
UNOS: The United Network for Organ Sharing
USRDS: The United States Renal Data System
WIT: Warm ischemic time
