# Supplementary material for: What is the impact of human leukocyte antigen mismatching on graft survival and mortality in renal transplantation? A meta-analysis of 23 cohort studies involving 486,608 recipients
Source: BMC Nephrol. 2018 May 18;19:116. doi: 10.1186/s12882-018-0908-3 (PMC5960106; doi:10.1186/s12882-018-0908-3)
Supplement: Supplementary file 1 — Supplemental Methods. (DOCX 45 kb) [file 12882_2018_908_MOESM1_ESM.docx]

**Additional file 3 - Supplemental Methods**

**STUDY PROTOCOL First drafted in Dec 2016**

**Objectives and aims:** We aimed to evaluate the associations between HLA mismatching and graft failure and mortality of adult kidney transplantation recipients in the era of modern immunosuppressive therapy.

**Background:** Compared with dialysis, kidney transplantation is recognized as a better choice for end-stage renal disease (ESRD) [1]. In the report of global database on donation and transplantation (www.transplant-observatory.org), about 80,000 kidney transplantations were operated every year globally [2]. Human leukocyte antigens (HLA) histocompatibility is an essential biological factor that affects the prognosis of kidney transplantation [3]. In many countries it is of importance in making allocation policies of scarce donor organs to recipients, based on the degree of HLA-mismatching [4]. With the application of more potent immunosuppression, the success rates of kidney transplantation increased markedly [5]. It seems that the prognostic values of HLA compatibility have been attenuated. Moreover, the magnitude of the effect of HLA mismatching on the outcome of kidney transplantation is controversial [6]. Extensive cohort studies reported the associations of HLA mismatching with outcomes of kidney transplantation [7-20], but the reported data were inconsistent and to date comprehensive meta-analysis of the available evidence is lacking. In the meta-analysis we aimed to evaluate the magnitude effect of HLA mismatching on the long-term survival outcomes of kidney transplantation.

**Research plan:**

1. **Methods of review**

This meta-analysis followed the Meta-analysis of Observational Studies in Epidemiology (MOOSE) reporting guidelines and the Preferred Reporting Items for Systematic Reviews and Meta-Analysis (PRISMA) Checklist.

1. **Data sources:**

Relevant studies were searched comprehensively by searching PubMed, EMBASE, and the Cochrane Library database from inception to 31 December 2016 without language restriction, using the following combinations of Medical Subject Headings (MeSH) and text words: kidney transplantation, HLA, and all known spelling of survival.

1. **Study selection:**

Type of studies:

Randomized controlled trials, cohort studies and case controlled researches, study the effect of human leukocyte antigen mismatching on outcomes of primary kidney transplantation.

Type of participants:

Inclusion criteria:

1. had a patient population comprising adult kidney transplant recipients
2. a cohort study or trial that reported associations between HLA mismatching and kidney transplantation
3. provided the hazard ratio (HR) and its 95% confidence interval (CI) or standard errors for graft failure or mortality associated with HLA or subtype mismatching degree

Exclusion criteria:

1. Case reports, reviews and other articles without original data
2. Trials without reference to primary kidney transplantation
3. Trials without the intervening of HLA matching or mismatching
4. Trials without outcomes of patient survival, graft survival.
5. **Type of outcome measures:**

Primary clinical endpoints were effect of HLA mismatching on overall mortality, death-censored graft failure and graft failure; secondary endpoints were effect of HLA subtype (HLA-DR/HLA-A/HLA-B) mismatching on overall mortality, graft failure and death-censored graft failure.

**Search strategy**

**PUBMED**

1. Kidney Transplantation [MeSH]
2. (kidney or renal) and (allograft* or transplant* or graft*).tiab
3. or/1-2
4. Histocompatibility Antigens [MeSH]
5. Histocompatibility.tiab
6. HLA.tiab
7. human leukocyte antigen.tiab
8. major histocompatibility complex.tiab
9. MHC.tiab
10. or/4-9
11. match*.tiab
12. mismatch*.tiab
13. typ*.tiab
14. compatib*.tiab
15. or/11-14
16. Mortality [MeSH]
17. Mortalit*.tiab
18. Death*.tiab
19. Kaplan meier.tiab
20. Proportional hazard*.tiab
21. Survival*.tiab
22. Or/16-21
23. Humans
24. Animals
25. And/23,24
26. 24 NOT 25
27. 23 NOT 26
28. and/3,10,15,22,27

**EMBASE**

1. exp Kidney Transplantation/
2. (kidney or renal) and (allograft* or transplant* or graft* or recipient*).tiab
3. or/1-2
4. exp Histocompatibility Antigens/
5. Histocompatibility
6. HLA
7. human leukocyte antigen
8. major histocompatibility complex
9. MHC
10. or/4-9
11. match*.tiab
12. mismatch*.tiab
13. typ*.tiab
14. compatib*.tiab
15. or/11-14
16. exp Mortality/
17. exp Proportional Hazard Model/
18. exp Kaplan meier method/
19. exp Survival/
20. exp Survival Analysis/
21. mortalit*.tiab
22. death*.tiab
23. survival*.tiab
24. proportional NEAR/1 hazard*.tiab
25. Kaplan meier.tiab
26. Hazard NEAR/1 (model* or ratio*).tiab
27. Or/16-26
28. Humans
29. Animals
30. And/28,29
31. 29 NOT 30
32. 28 NOT 31
33. and/3,10,15.27.32

**COCHRANE CONTROLLED TRIALS**

1. MeSH descriptor Kidney Transplantation explode all trees
2. (kidney or renal) and (transplant* or allograft* or graft* or recipient*)
3. 1 or 2
4. MeSH descriptor Histocompatibility Antigens explode all trees
5. Histocompatibility.tw
6. HLA.tw
7. human leukocyte antigen.tw
8. major histocompatibility complex.tw
9. MHC.tw
10. 4 or 5 or 6 or 7 or 8 or 9
11. match*.tw
12. mismatch*.tw
13. compatibl*.tw
14. 11 or 12 or 13
15. 3 and 10 and 14

**Reference**

1. Ferrari P, Weimar W, Johnson RJ, Lim WH, Tinckam KJ. Kidney paired donation: principles, protocols and programs. Nephrol Dial Transplant. 2015; 30(8):1276-85
2. Global Observatory on Donation and Transplantation, World Health Organization. Organ donation and transplantation activities 2014. http://www.transplant-observatory.org/data-reports-2014/. Accessed April, 2016
3. Al-Otaibi T, Gheith O, Mosaad A, Nampoory MR, Halim M, Said T, et al. Human leukocyte antigen-DR mismatched pediatric renal transplant: patient and graft outcome with different kidney donor sources. Experimental and clinical transplantation: official journal of the Middle East Society for Organ Transplantation. 2015; 13 Suppl 1:117-23
4. Ferrari P, Weimar W, Johnson RJ, Lim WH, Tinckam KJ. Kidney paired donation: principles, protocols and programs. Nephrol Dial Transplant. 2015; 30(8):1276-85
5. Stang A. Critical evaluation of the Newcastle–Ottawa Scale for the assessment of the quality of nonrandomized studies in meta-analyses. Eur J Epidemiol. 2010; 25: 603–5
6. [Opelz G](https://www.ncbi.nlm.nih.gov/pubmed/?term=Opelz%20G%5BAuthor%5D&cauthor=true&cauthor_uid=24461002), [Döhler B](https://www.ncbi.nlm.nih.gov/pubmed/?term=D%C3%B6hler%20B%5BAuthor%5D&cauthor=true&cauthor_uid=24461002). Ceppellini Lecture 2012: collateral damage from HLA mismatching in kidney transplantation. Tissue Antigens. 2013;82(4):235-42.
7. de Fijter JW, Mallat MJ, Doxiadis, II, Ringers J, Rosendaal FR, Claas FH, et al. Increased immunogenicity and cause of graft loss of old donor kidneys. JASN. 2001; 12(7):1538-46
8. Roodnat JI, van Riemsdijk IC, Mulder PG, Doxiadis I, Claas FH, JN IJ, et al. The superior results of living-donor renal transplantation are not completely caused by selection or short cold ischemia time: a single-center, multivariate analysis. Transplantation. 2003; 75(12):2014-8
9. Tekin S, Yavuz HA, Yuksel Y, Yucetin L, Ates I, Tuncer M, et al. Kidney Transplantation From Elderly Donor. Transplant Proc. 2015; 47(5):1309-11
10. Mandal AK, Snyder JJ, Gilbertson DT, Collins AJ, Silkensen JR. Does cadaveric donor renal transplantation ever provide better outcomes than live-donor renal transplantation? Transplantation. 2003; 75(4):494-500
11. Arias LF, Blanco J, Sanchez-Fructuoso A, Prats D, Duque E, Saiz-Pardo M, et al. Histologic assessment of donor kidneys and graft outcome: multivariate analyses. Transplant Proc. 2007; 39(5):1368-70
12. Cho H, Yu H, Shin E, Kim YH, Park SK, Jo MW. Risk Factors for Graft Failure and Death following Geriatric Renal Transplantation. PloS one. 2016;11(4): e0153410
13. Gomez EG, Hernandez JP, Lopez FJ, Garcia JR, Montemayor VG, Curado FA, et al. Long-term allograft survival after kidney transplantation. Transplant Proc. 2013; 45(10):3599-602
14. Croke R, Lim W, Chang S, Campbell S, Chadban S, Russ G, et al. HLA-mismatches increase risk of graft failure in renal transplant recipients initiated on cyclosporine but not tacrolimus. Nephrology. 2010; 15:38
15. Laging M, Kal-van Gestel JA, van de Wetering J, Ijzermans JN, Weimar W, Roodnat JI. The relative importance of donor age in deceased and living donor kidney transplantation. Transpl Int. 2012; 25(11):1150-57.
16. Asderakis A, Dyer P, Augustine T, Worthington J, Campbell B, Johnson RW. Effect of cold ischemic time and HLA matching in kidneys coming from "young" and "old" donors: do not leave for tomorrow what you can do tonight. Transplantation. 2001; 72(4):674-8
17. Schnuelle P, Lorenz D, Mueller A, Trede M, Van Der Woude FJ. Donor catecholamine use reduces acute allograft rejection and improves graft survival after cadaveric renal transplantation. Kidney Int. 1999; 56(2):738-46.
18. Connolly JK, Dyer PA, Martin S, Parrott NR, Pearson RC, Johnson RW. Importance of minimizing HLA-DR mismatch and cold preservation time in cadaveric renal transplantation. Transplantation. 1996; 61(5):709-14
19. Hariharan S, McBride MA, Cherikh WS, Tolleris CB, Bresnahan BA, Johnson CP. Post-transplant renal function in the first year predicts long-term kidney transplant survival. Kidney Int. 2002; 62(1):311-8
20. Cho YW, Lemp N, Shah T, Sampio MS, Hutchinson IV, Cicciarelli J. Long-term risk factors of kidney graft survival in the modern immunosuppression era. Am J Transplant. 2012; 12:490
