# Supplementary material for: What is the impact of human leukocyte antigen mismatching on graft survival and mortality in renal transplantation? A meta-analysis of 23 cohort studies involving 486,608 recipients
Source: BMC Nephrol. 2018 May 18;19:116. doi: 10.1186/s12882-018-0908-3 (PMC5960106; doi:10.1186/s12882-018-0908-3)
Supplement: Supplementary file 2 — Table S1. The MOOSE checklist. (DOCX 103 kb) [file 12882_2018_908_MOESM2_ESM.docx]

| Table S1: The MOOSE checklist | Criteria | Brief description of how the criteria were handled in the meta-analysis |
| --- | --- | --- |
|  | **Reporting of background should include** |  |
| 🗸 | Problem definition | Shortage of donor organ is becoming an increasing challenge worldwide, partly due to high demand for donor-recipient human leukocyte antigen (HLA) compatibility. Current evidence of the effect of HLA mismatching on the outcomes of kidney transplantation remains controversial (especially in the era of new potent immunosuppression protocols) |
| 🗸 | Hypothesis statement | HLA mismatching may increase risk of adverse postoperative outcomes (mortality, graft loss, rejection, etc) |
| 🗸 | Description of the study outcomes | Graft failure, mortality, graft rejection |
| 🗸 | Types of exposure or intervention | Kidney Transplantation |
| 🗸 | Type of study designs used | Cohort studies were included. |
| 🗸 | Study population | We included adult recipients that evaluated HLA/sub-types and the risk of adverse postoperative outcomes |
|  | **Reporting of search strategy should include** |  |
| 🗸 | Qualifications of searchers | The credentials of all reviewers are shown in author list. |
| 🗸 | Search strategy, including time period included in the synthesis and keywords | Two investigators conducted the literature search from the inception of the databases to December, 2016. Any discrepancies in inclusion were resolved through discussions with a third reviewer. |
| 🗸 | Databases and registries searched | PubMed/ EMBASE/Cochrane Library |
| 🗸 | Search software used, name and version, including special features | No specific search software was used. EndNote X7.0 was used to merge retrieved citations and eliminate duplications. |
| 🗸 | Use of hand searching | We contact the large libraries home and abroad to request or buy the articles as comprehensive as possible. We hand searched bibliographies of retrieved papers for additional references. |
| 🗸 | List of citations located and those excluded, including justifications | Details of the literature review process are outlined in Figure 1 of the article. |
| 🗸 | Method of addressing articles published in languages other than English | We searched the literature without language restriction, and articles published in languages other than English were translated into English or Chinese by friends who work as translators. |
| 🗸 | Method of handling abstracts and unpublished studies | We contacted the authors to make clarifications regarding their published manuscript. |
| 🗸 | Description of any contact with authors | Mainly by E-mails. In some time by Telephone or Letters. |
|  | **Reporting of methods should include** |  |
| 🗸 | Description of relevance or appropriateness of studies assembled for assessing the hypothesis to be tested | Specific inclusion and exclusion criteria following PICOTS format were described in the methods section. |
| 🗸 | Rationale for the selection and coding of data | Data extractions from each eligible study (included the first author’s name, publication year, location, study population, sample size, male proportion, mean age, sex, median follow-up, donor source, data source etc.). |
| 🗸 | Assessment of confounding | Sensitivity analysis, predefined subgroup analysis and univariate meta-regression were assessed to address potential confounding. |
| 🗸 | Assessment of study quality, including blinding of quality assessors; stratification or regression on possible predictors of study results | The methodological quality of included studies was described using the Newcastle-Ottawa Scale (NOS). A stratified analysis based on the quality were performed and reported in Results section. |
| 🗸 | Assessment of heterogeneity | The heterogeneity was assessed by using chi-square test and the I^2^-statistic. A value of I^2^ of 0-50% represents low heterogeneity, 50-75% moderate heterogeneity and >75% high heterogeneity |
| 🗸 | Description of statistical methods in sufficient detail to be replicated | Description of statistical methods, sensitivity analyses, random effects univariate meta-regression and assessment of publication bias, are provided in the Methods section. |
| 🗸 | Provision of appropriate tables and graphics | Two tables (Included Studies Characteristics) and 5 figures were provided main findings. |
|  | **Reporting of results should include** |  |
| 🗸 | Graph summarizing individual study estimates and overall estimate | Mainly in Figure 2-5 |
| 🗸 | Table giving descriptive information for each study included | Table 1 |
| 🗸 | Results of sensitivity testing | In the part of “results” |
| 🗸 | Indication of statistical uncertainty of findings | 95% CI and I^2^ statistic were reported |
|  | **Reporting of discussion should include** |  |
| 🗸 | Quantitative assessment of bias | Funnel plots and Egger’s test were performed to evaluate publication bias. |
| 🗸 | Justification for exclusion | Studies that provided only abstracts were excluded. |
| 🗸 | Assessment of quality of included studies | Quality of included studies was described in the first paragraph of Results sections, all studies were of high quality (NOS>6). |
