# Supplementary material for: What is the impact of human leukocyte antigen mismatching on graft survival and mortality in renal transplantation? A meta-analysis of 23 cohort studies involving 486,608 recipients
Source: BMC Nephrol. 2018 May 18;19:116. doi: 10.1186/s12882-018-0908-3 (PMC5960106; doi:10.1186/s12882-018-0908-3)
Supplement: Supplementary file 4 — Table S3. Newcastle-Ottawa Scale (NOS) score for evaluation of study quality. (DOCX 73 kb) [file 12882_2018_908_MOESM4_ESM.docx]

| **First author,**  Table S3: Newcastle-Ottawa Scale (NOS) score for evaluation of study quality  **published year** | **Study Design** | **Assessment of**  **confounding** | **Exposure definition** | **Method of**  **outcome assessment** | **Follow-up time** | **Total score** | **Quality of study** |
| --- | --- | --- | --- | --- | --- | --- | --- |
| De Fijter (2001) | 2 | 2 | 1 | 2 | 1 | 8 | High |
| Roodnat (2003) | 2 | 2 | 1 | 2 | 1 | 8 | High |
| Tekin (2015) | 2 | 1 | 1 | 2 | 1 | 7 | High |
| Mandal (2003) | 2 | 2 | 1 | 2 | 1 | 8 | High |
| Arias (2007) | 2 | 2 | 1 | 2 | 1 | 8 | High |
| Cho (2016) | 2 | 2 | 1 | 2 | 1 | 8 | High |
| Gomez (2013) | 2 | 2 | 1 | 2 | 1 | 8 | High |
| Laging (2012) | 2 | 2 | 1 | 2 | 1 | 8 | High |
| Laging (2014) | 2 | 2 | 1 | 2 | 1 | 8 | High |
| Schnuelle (1999) | 2 | 2 | 1 | 2 | 1 | 8 | High |
| Hariharan (2002) | 1 | 2 | 1 | 2 | 1 | 7 | High |
| Massie (2016) | 2 | 2 | 1 | 2 | 1 | 8 | High |
| Cho (2012) | 2 | 2 | 1 | 2 | 1 | 8 | High |
| Croke (2010) | 2 | 2 | 1 | 2 | 1 | 8 | High |
| Connolly (1996) | 2 | 2 | 1 | 2 | 1 | 8 | High |
| Asderakis (2001) | 2 | 2 | 1 | 2 | 1 | 8 | High |
| Opelz (2007) | 2 | 2 | 1 | 2 | 1 | 8 | High |
| Amatya (2010) | 2 | 2 | 1 | 2 | 1 | 8 | High |
| Zukowski (2014) | 2 | 0 | 1 | 2 | 1 | 6 | High |
| Fellstrom (2005) | 2 | 0 | 1 | 2 | 1 | 6 | High |
| Summers (2010) | 1 | 1 | 1 | 2 | 1 | 6 | High |
| Van (1996) | 2 | 2 | 1 | 2 | 1 | 8 | High |
| Lynch (2013) | 2 | 2 | 1 | 2 | 1 | 8 | High |
