# Supplementary figures and images for: What is the impact of human leukocyte antigen mismatching on graft survival and mortality in renal transplantation? A meta-analysis of 23 cohort studies involving 486,608 recipients
Source: BMC Nephrol. 2018 May 18;19:116. doi: 10.1186/s12882-018-0908-3 (PMC5960106; doi:10.1186/s12882-018-0908-3)

**A: B:**

**
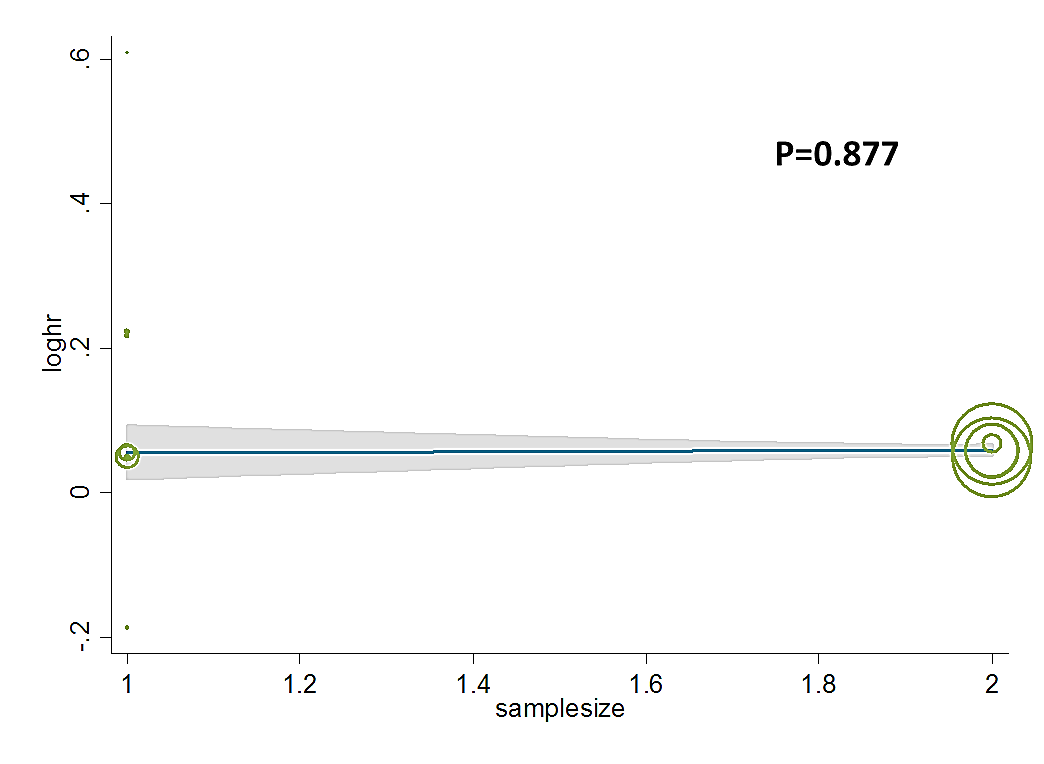

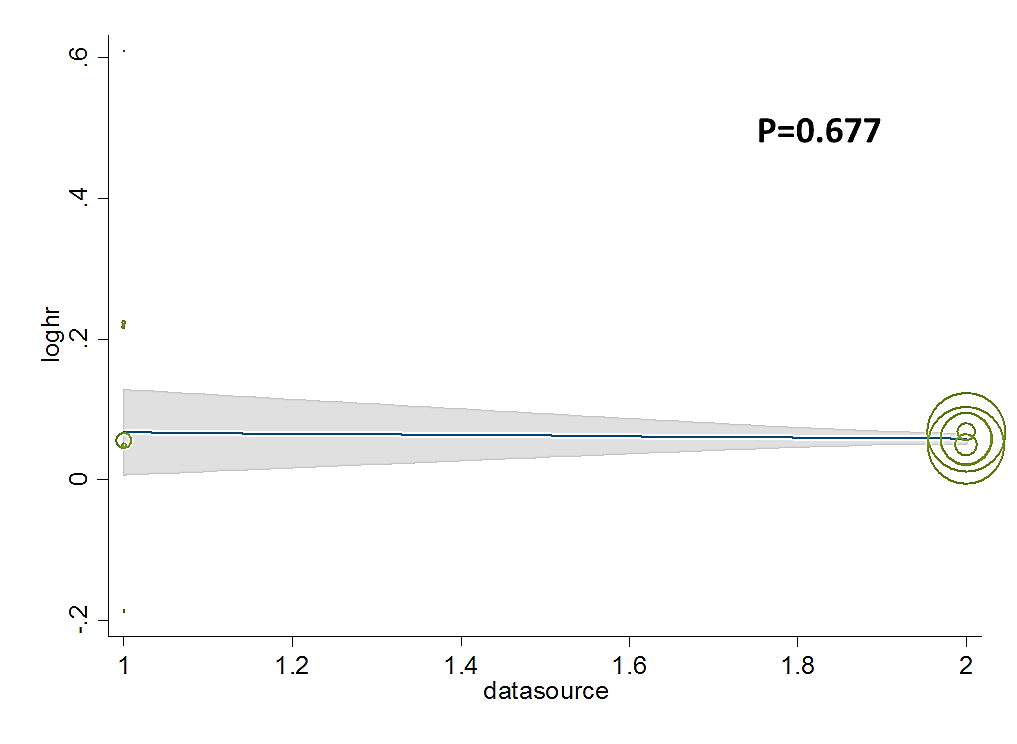
**

**C: D:**

**
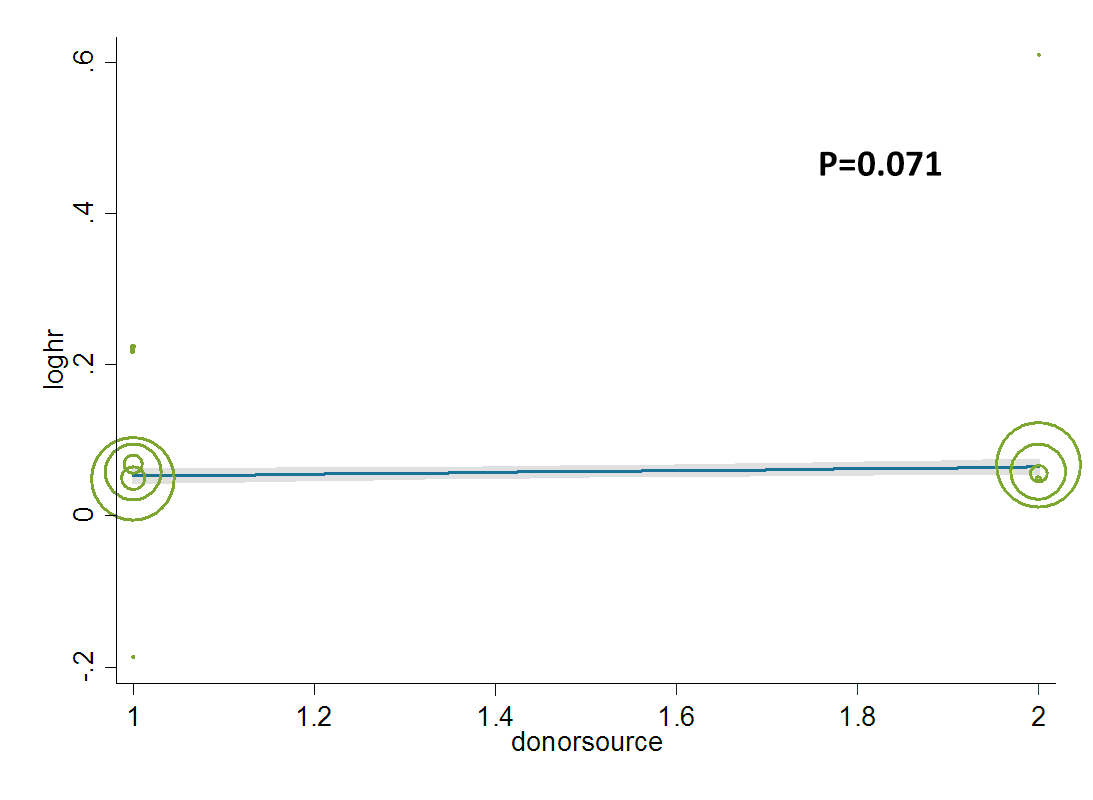

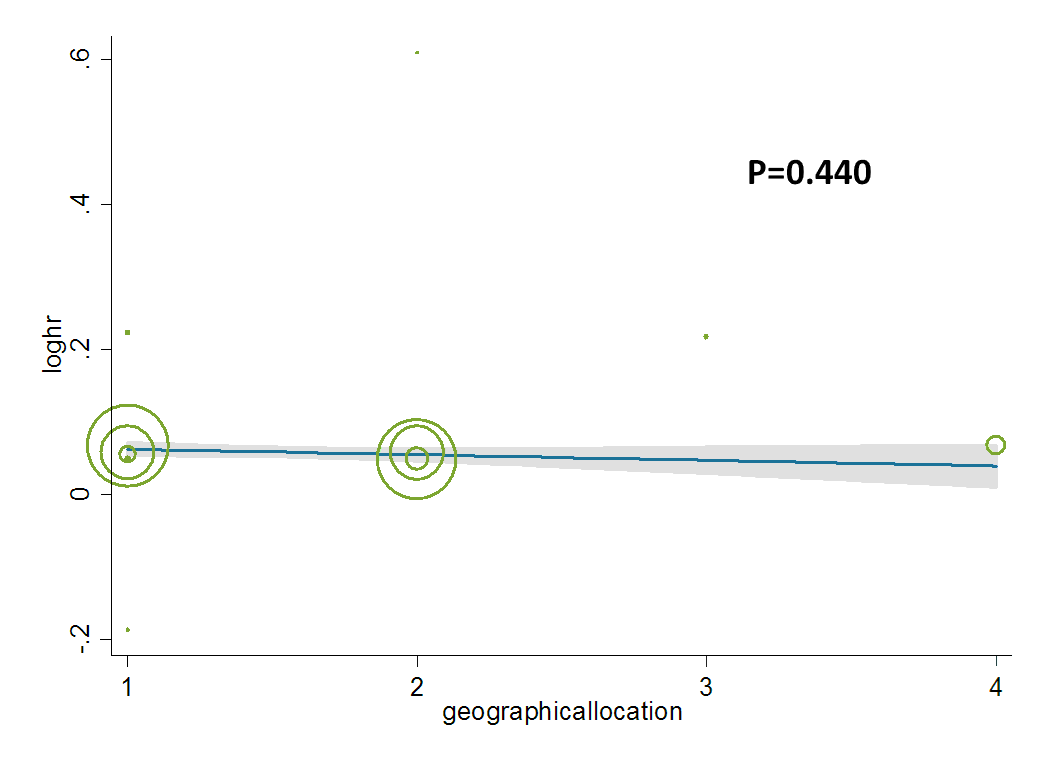
**

Supplement: Supplementary file 5 — Figure S1. Meta-regression of HLA mismatches on graft failure for primary determinant confounders (A: Sample size; B: Data source; C: Donor source; D: Geographical locations). (DOCX 608 kb) [file 12882_2018_908_MOESM5_ESM.docx]

**A: B:**

**
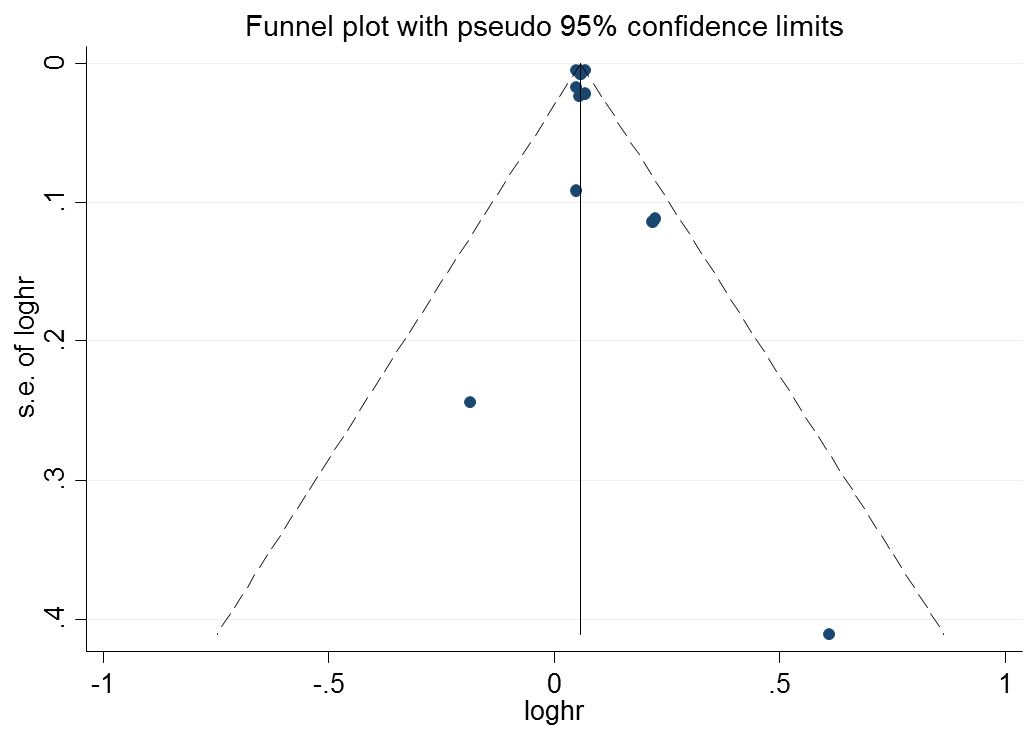

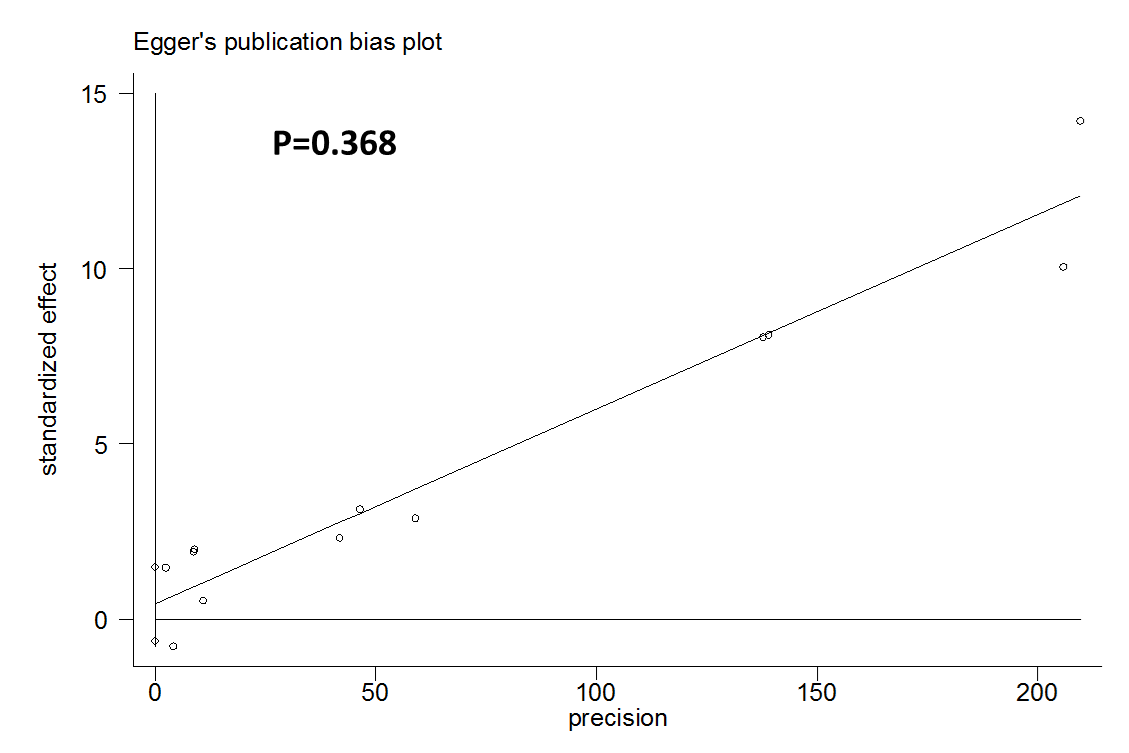
**

Supplement: Supplementary file 6 — Figure S2. Funnel plot and Egger test for publication bias among studies that evaluated association HLA per mismatch and overall graft failure. (A: Funnel plot; B: Egger test). (DOCX 81 kb) [file 12882_2018_908_MOESM6_ESM.docx]

**
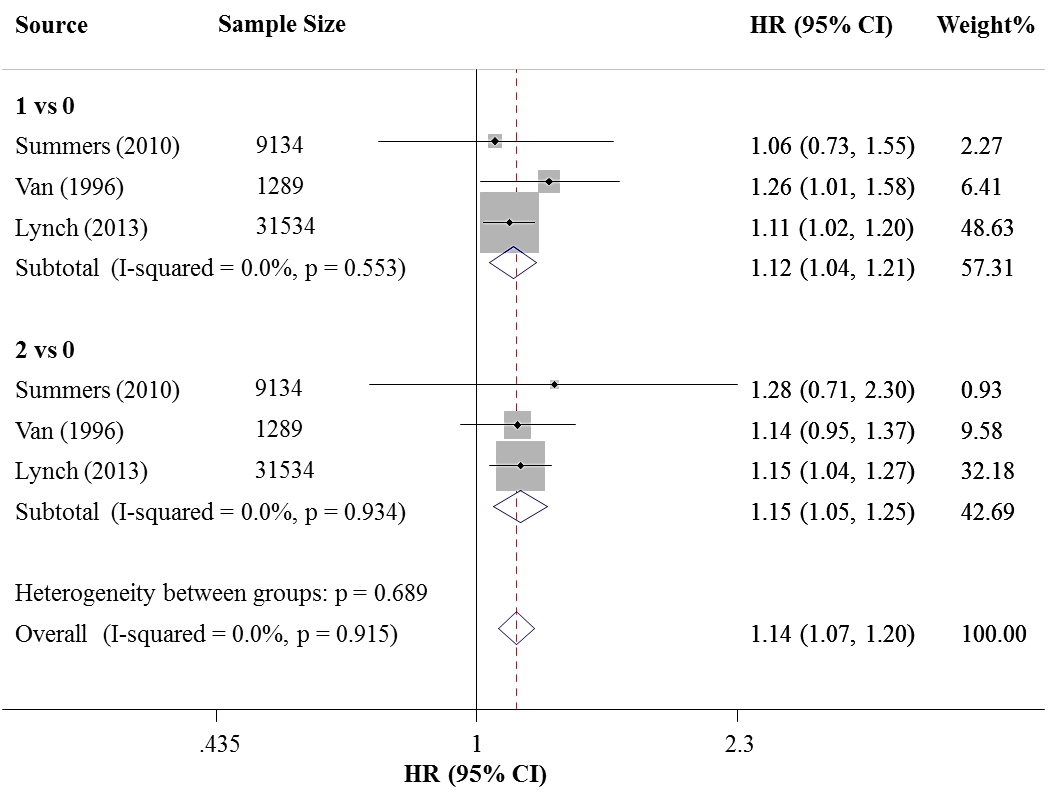
**

Supplement: Supplementary file 7 — Figure S3. Forest plot that evaluated the impact of 1 or 2 HLA-DR mismatches versus 0 mismatches on overall graft failure. (DOCX 587 kb) [file 12882_2018_908_MOESM7_ESM.docx]

**A:**

**
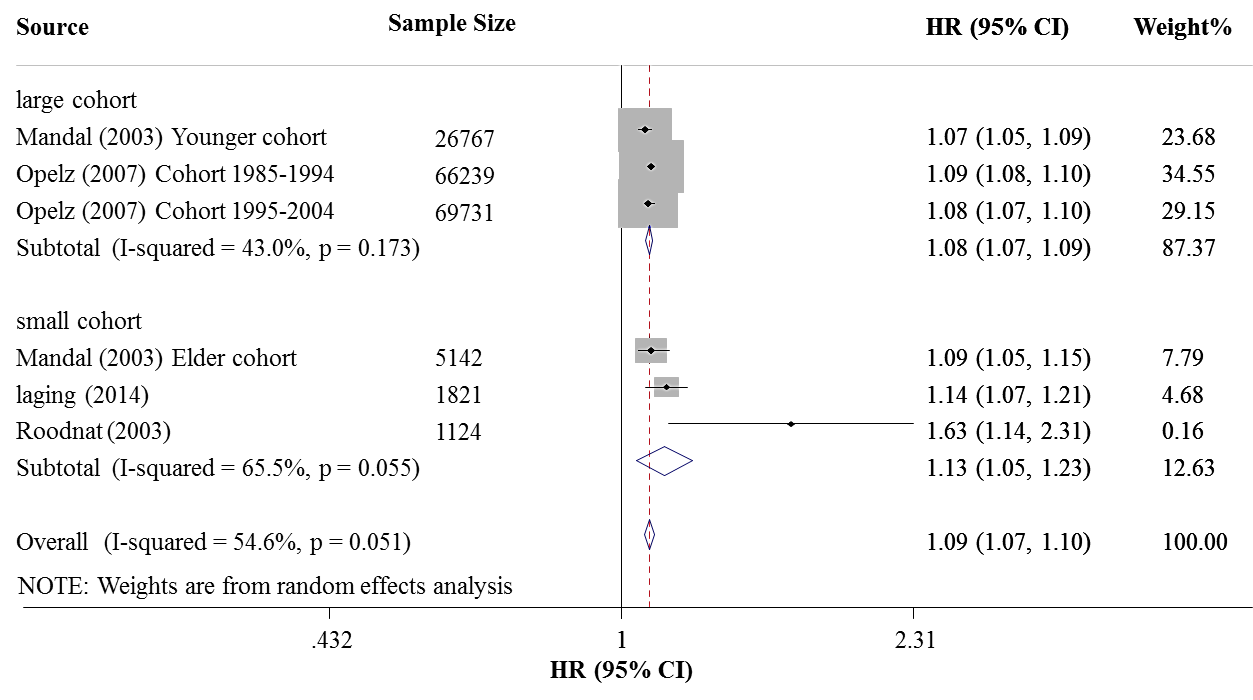
**

**B:**

**
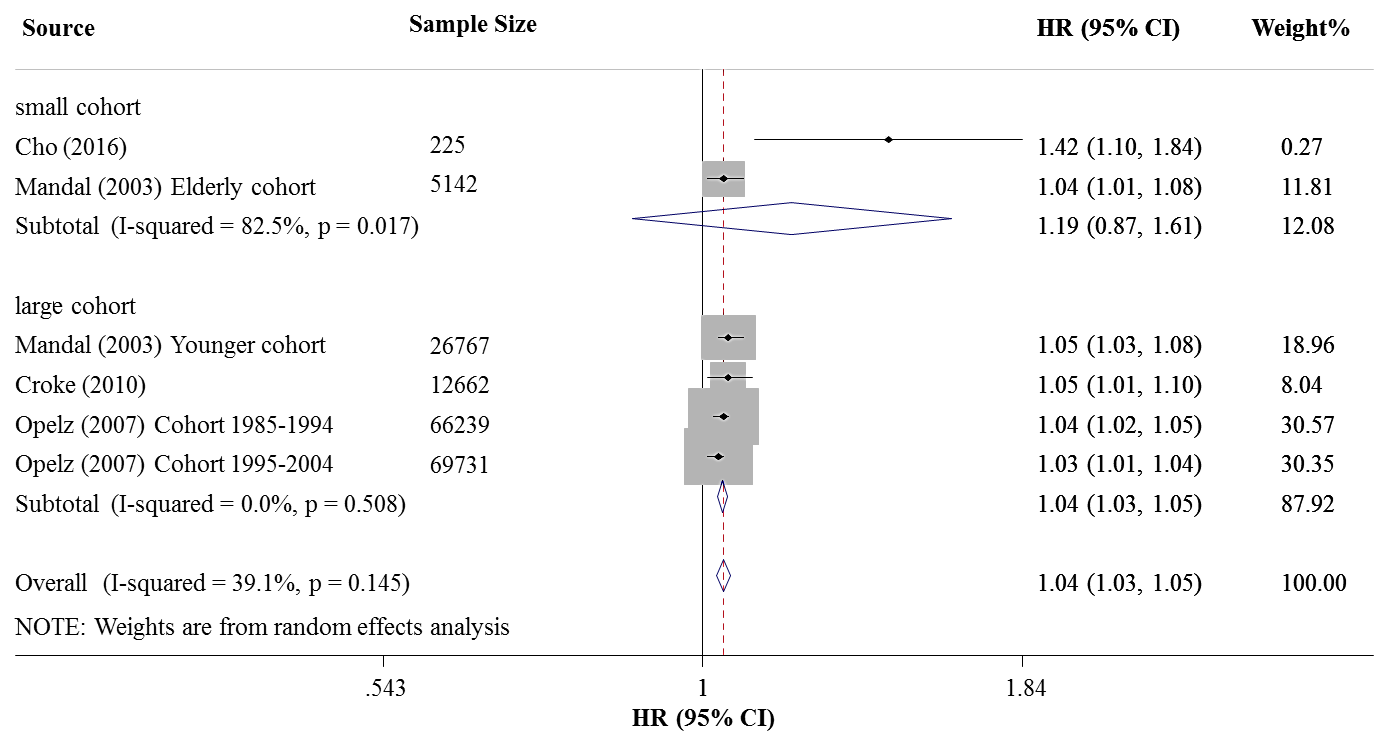
**

Supplement: Supplementary file 8 — Figure S4. Forest plot after stratification for sample size (≥10,000 vs < 10,000) of cohorts, to evaluate association between (A) HLA per mismatch and death-censored graft failure; (B) HLA per mismatch and all-cause mortality. (DOCX 1230 kb) [file 12882_2018_908_MOESM8_ESM.docx]
